# Combining Evolutionary Steering and Coevolutionary Phage Training to Generate Predictable Phage-Antibiotic Synergy

**DOI:** 10.1038/s44259-026-00249-w

**Published:** 2026-07-14

**Authors:** Nehme Lahoud, Sweetzel D. Labador, Mrudula Sane, Divya Ram, Gabriel E. Diaz, Justin R. Meyer

**Affiliations:** Ecology, Behavior and Evolution Department, University of California San Diego, San Diego, CA 92093 USA

**Keywords:** Computational biology and bioinformatics, Microbiology

## Abstract

The rapid rise of multidrug-resistant bacterial pathogens has renewed interest in bacteriophage therapy, yet bacterial resistance to phages can evolve rapidly. Two evolutionary strategies have been proposed to address this challenge: coevolutionary phage training, which adapts phages to anticipated resistance mechanisms, and phage-antibiotic synergy, which exploits tradeoffs between phage resistance and antibiotic susceptibility. Here, we show that these strategies intersect in complex but predictably exploitable ways. Using *Escherichia coli* and bacteriophage λ, we demonstrate that resistance to a trained—but not an untrained—phage generates collateral sensitivity to erythromycin and rifampicin, but not to five other antibiotics. This training-induced synergy is mechanistically linked to repeated disruption of *lpcA*, a gene involved in lipopolysaccharide core biosynthesis. Leveraging this mechanistic insight, we successfully predicted that novobiocin and rifapentine would also exhibit collateral sensitivity with a trained λ phage. We further show that incorporating sub-lethal erythromycin during phage training reshapes coevolutionary trajectories, increasing the frequency with which phages evolve the ability to use a second host receptor and thereby enhancing bacterial suppression. Together, these results reveal that despite the inherent complexity of phage-bacteria coevolution, key outcomes can be anticipated and directed. Our findings highlight the promise of evolution-informed, predictive strategies for designing therapies.

## Introduction

Antibiotic resistance is accelerating at a pace that threatens to render current drugs unusable, as multidrug-resistant pathogens increasingly compromise standard-of-care treatments worldwide^1–4^. This crisis has prompted urgent searches for alternative antimicrobial strategies. Among the most promising are bacteriophages—viruses that specifically infect and kill bacteria—which have recently re-emerged as viable therapeutics after nearly a century of dormancy in Western medicine^5,6^. Phage therapy has produced promising successes in compassionate-use settings, including the clearance of otherwise untreatable *Acinetobacter*, *Pseudomonas*, *Mycobacterium*, and other life-threatening infections, and several ongoing clinical trials are now evaluating its broader safety and efficacy^7–9^. Unlike antibiotics, phages are expected to replicate at the site of infection, amplifying precisely where the patient needs them most, while targeting pathogens with high specificity and permitting individualized treatment designs^10^. These characteristics have generated widespread enthusiasm for deploying phages as frontline alternatives when antibiotics fail.

Yet phage therapy faces a fundamental challenge: the same evolutionary processes that produced the antibiotic resistance crisis also operate against phages. Bacteria and phages have been engaged in a coevolutionary arms race for billions of years, cycling through rapid rounds of defense acquisition, counter-defense innovation, and escape^11^. As a result, bacteria often evolve resistance to phages extraordinarily quickly, rendering this approach ineffective in many instances^12–15^. Unlike antibiotic deployment, where resistance evolution was addressed reactively after drugs were deployed, phage therapy offers a new opportunity to address the resistance problem proactively before large-scale clinical use. Because resistance is fundamentally an evolutionary problem, it makes sense to leverage evolutionary principles to engineer more resilient therapeutics.

Two evolutionary strategies have emerged to meet this challenge: coevolutionary phage training exposes phages to their bacterial host over extended periods, allowing them to accumulate counter-defenses, effectively stockpiling evolutionary “ammunition” by adapting to a range of resistance mechanisms the host population can generate. Through these prolonged cycles of attack and counterattack, trained phages often evolve improved adsorption, expanded receptor use, and enhanced suppression of emerging resistance^16–22^. Phage evolutionary steering, by contrast, leverages known interactions between phage resistance and antibiotic susceptibility, or phage-antibiotic synergies, to bias bacterial evolutionary escape into maladaptive directions^23,24^. For instance, phage resistance has been shown to frequently arise through mutations in lipopolysaccharide (LPS) biosynthesis genes, which can alter the composition of the outer membrane and increase permeability to certain antibiotics^25^. Likewise, mutations in TolC, the outer-membrane channel of the *Escherichia coli* AcrAB-TolC multidrug efflux pump, block adsorption of phages that use TolC as a receptor while simultaneously impairing antibiotic efflux, lowering minimum inhibitory concentrations for several antibiotics, and creating predictable collateral sensitivity^24,26–28^. Steering exploits these tradeoffs by biasing bacterial evolutionary escape routes into resistance pathways that carry increased vulnerability to the antibiotic, thereby restricting the emergence of dual resistance.

Each strategy, however, has limitations. For coevolutionary phage training, even well-trained phages have been shown to ultimately be escaped by bacteria that acquire multi-step, costly, yet effective resistance mutations^18,19^. Additionally, results can be dependent on the specific phage-host pair and may not necessarily extend beyond those specific interactions, making phage training potentially less likely to be effective when bacterial isolates from one patient are used to train phage to treat another. As for evolutionary steering, identifying phage–antibiotic pairs that produce collateral sensitivity is challenging due to the difficult-to-predict nature of evolutionary responses and often requires extensive screening with frequent failures. Additionally, while some phage resistance mutations produce synergistic tradeoffs in certain systems, they can also have the opposite effect^29–32^. For example, Burmeister et al. demonstrated that mutations in TolC and LPS evolved for phage resistance also cause collateral sensitivity to antibiotics, but certain LPS mutations can have the opposite effect and cause cross-resistance to the phage and antibiotics^26^. Together, these results point to the difficulty of finding pairs of phages and antibiotics that exhibit reliable tradeoffs.

To address these limitations and further enhance bacterial killing, it is natural to ask whether these two strategies can be combined. We propose a mechanistic rationale for expecting synergy. Resistance to coevolutionarily trained phages often carries substantial fitness costs above and beyond those associated with resistance to untrained phages. These increased penalties—such as constrained energy budgets, impaired stress-response and repair pathways, or compromised envelope integrity—are also likely to limit antibiotic tolerance. We therefore hypothesized that coevolutionary phage training preferentially selects resistance mutations that generate collateral antibiotic sensitivity, yielding a second benefit of training: training-induced antibiotic synergy. This framework provides a principled route to generating phage-antibiotic synergies, rather than relying on fortuitous combinations discovered by chance.

To evaluate this, we compared the antibiotic susceptibility of *E. coli* isolates evolving resistance to trained versus untrained phage λ and found that resistance to the trained phage was associated with increased sensitivity to two of seven antibiotics tested, indicating that phage training can induce collateral sensitivity. Comparative analysis of which antibiotics exhibited training-induced synergy enabled us to successfully predict a third antibiotic with this behavior, demonstrating the predictive power of this approach. These findings prompted us to ask whether the presence of antibiotics would alter which resistance mutations bacteria evolve, and if so, whether training should be conducted in the presence of antibiotics since phage therapy in clinical settings is administered alongside standard-of-care antibiotics. We therefore conducted coevolutionary phage training in the presence of sub-lethal antibiotic concentrations and found that antibiotics can redirect training trajectories in ways that enhance phage performance.

## Results

### Resistance to a trained phage can lead to collateral sensitivity

To test whether resistance to trained phage λ alters bacterial antibiotic susceptibility, we took advantage of materials from a previous study published by our group in which a range of resistant bacteria were isolated that evolved resistance to either untrained or trained phage isolates incapable of lysogeny^18^. We measured the half-maximal inhibitory concentration (IC50) of seven strains that differed in phage-resistance phenotype and/or evolutionary origins. Five strains spanning a range of resistance to the trained phage isolate were selected: Trn. Res. A and B originated from a coevolutionary training experiment where trained λ was cocultured with *E. coli* and engaged in an arms race; ‘A’ evolved partial resistance, and B gained complete resistance. The other three strains were generated from highly controlled Luria-Delbrück experiments where *E. coli* resistance mutations were selected using trained λ. Trn. Res. C and D harbor one and three mutations, respectively, conferring partial resistance to the trained phage and Trn. Res. E carries two mutations that grant complete resistance. To assess whether antibiotic susceptibility changes depend on whether the bacteriophage had been trained, we additionally included one strain fully resistant to untrained λ (Untr. Res.). Only one such strain was chosen because resistance to the untrained λ has been previously shown to predominantly evolve through a single type of mutation, *malT* loss-of-function mutations^33^. The final strain chosen for the IC50 assay was the λ-sensitive ancestor *E. coli* B REL606.

IC50 values were measured in the presence of seven antibiotics that ranged in modes of action and chemical properties. We expected that if trained phages select for resistance pathways that carry antibiotic vulnerabilities, then isolates resistant to trained λ should show lower IC50 values relative to untrained-λ-resistant and ancestral strains.

**Fig. 1:**
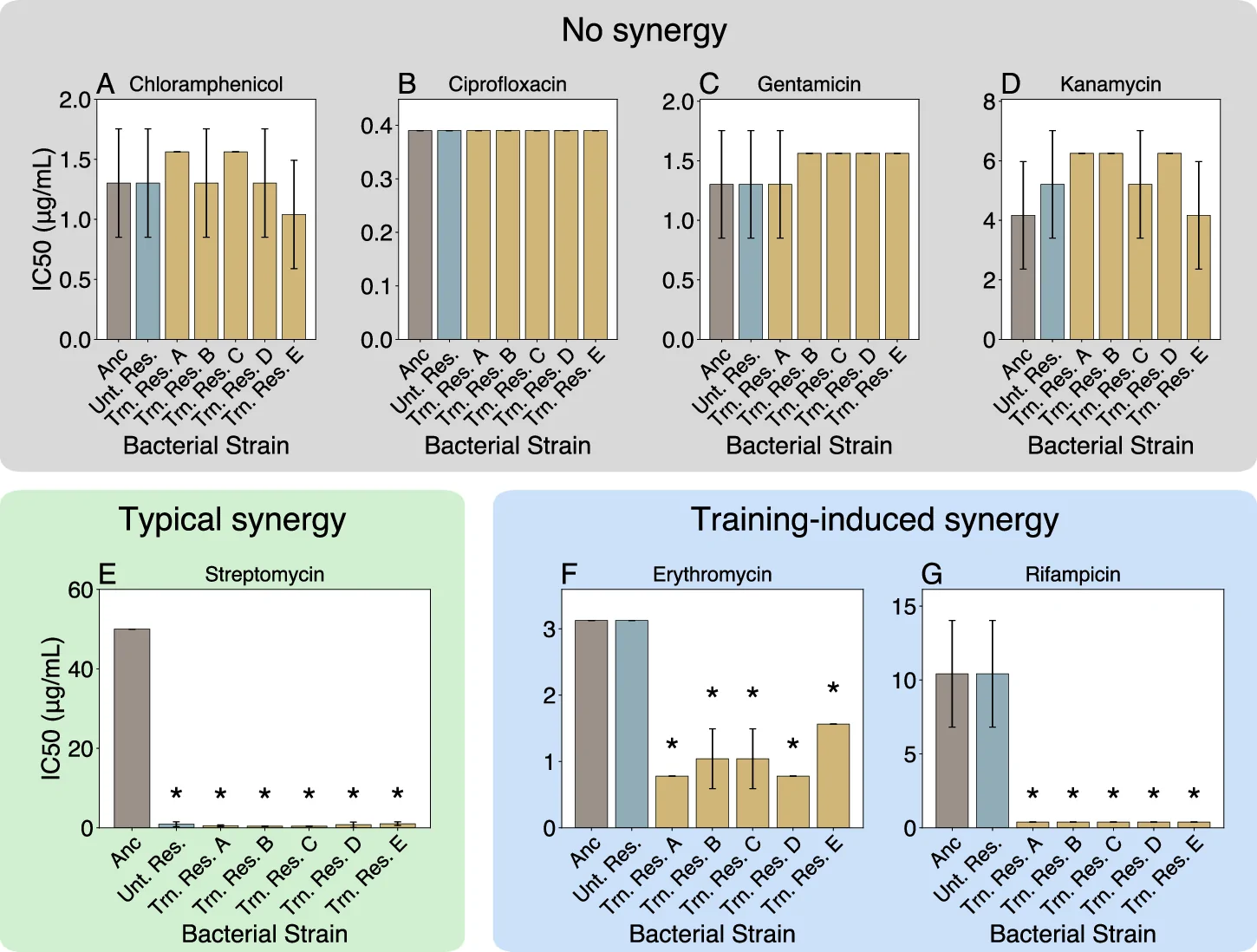
Susceptibility of *E. coli* strains with distinct bacteriophage λ resistance histories to seven antibiotics. Antibiotic responses fall into three qualitative categories: (1) no synergy, in which phage-resistant and phage-sensitive strains exhibit comparable antibiotic susceptibility; (2) typical synergy, in which resistance to any phage genotype increases antibiotic sensitivity; and (3) training-induced synergy, in which increased antibiotic sensitivity arises only in hosts that have evolved resistance to the trained phage, but not to an untrained phage. The *y*-axis indicates IC50, defined as the antibiotic concentration required to reduce bacterial growth by 50% after 24 h. IC50 values were measured for the λ-sensitive ancestral strain, a strain resistant to untrained λ, and five strains resistant to trained λ across seven antibiotics (*n* = 3 independent assays per strain). Bars represent mean IC50 values, and error bars indicate standard deviations. Asterisks denote statistically significant differences relative to the ancestral strain (Tukey’s post hoc test, *p* < 0.001).

In the chloramphenicol, ciprofloxacin, gentamicin, and kanamycin groups, no synergisms were observed, as evidenced by the consistent IC50 values for all strains examined (Fig. 1a–d). Typical phage-antibiotic synergy was observed in the streptomycin group, where strains resistant to either the untrained or trained phage showed lower IC50 values, i.e., higher antibiotic sensitivity (Fig. 1e) (one-way ANOVA: F(6,14) = 6190.204, *p* < 0.001; Tukey’s test). It had previously been found that the *E. coli* B strain REL606 does not evolve increased sensitivity to streptomycin over the course of laboratory evolution in the absence of phages, suggesting that the increased sensitivity we observed was likely induced by phage resistance evolution and not lab adaptation^34^. For erythromycin and rifampicin, all strains resistant to trained phage λ exhibited significantly reduced IC50 values, regardless of genotype, relative to both the untrained-phage-resistant strain and the ancestral REL606 strain, which showed comparable levels of antibiotic tolerance (Fig. 1f, g) (one-way ANOVA: erythromycin: *F*(6,14) = 56.706, *p* < 0.001; rifampicin: *F*(6,14) = 19.303, *p* < 0.001; Tukey’s test). In Fig. 1f, g, this pattern is referred to as *Training-induced Synergy*, as a case in which resistance to the trained phage specifically coincided with increased antibiotic sensitivity. Overall, no antagonistic interactions where phage resistance increases antibiotic resistance (cross-resistance) were observed.

These results indicate that resistance to trained phage λ can, as a byproduct, increase bacterial susceptibility to erythromycin and rifampicin. More broadly, they suggest that coevolutionary training may enable phages that are not typically compatible with antibiotic-based evolutionary steering to become viable therapeutic options.

### Mutations in the LPS core biosynthesis gene *lpcA* underlie training-induced antibiotic sensitivity

**Fig. 2:**
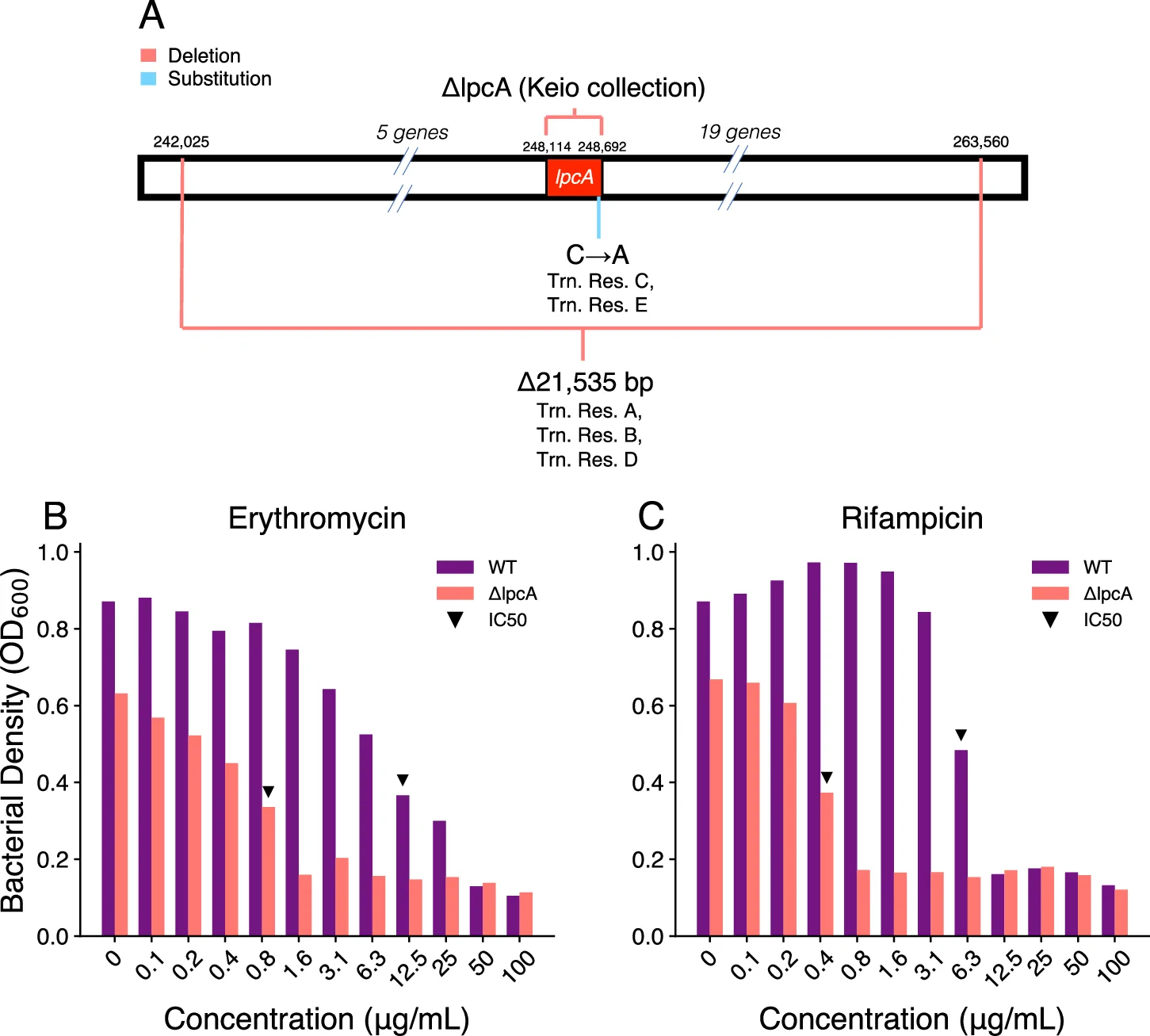
Mutations in the *lpcA* gene and their phenotypic effects. **A** Schematic depiction of *lpcA*-associated mutations in isolates that evolved resistance to trained phages and an engineered knockout strain. **B**, **C** Antibiotic sensitivity assays comparing an *lpcA* knockout strain (Δ*lpcA*) to its isogenic wild-type parent (K-12) across increasing concentrations of erythromycin (**B**) and rifampicin (**C**) (*n* = 6). The Δ*lpcA* strain exhibits a baseline growth deficit and a reduced IC50 relative to the wild type for both antibiotics.

To identify the genetic basis of the observed training-induced synergy, we examined the genomes of isolates that became more susceptible to erythromycin and rifampicin, searching for any shared mutations (mutation summary available in Supplementary Information in Borin et al.^18^). Genomic analysis revealed that all isolates exhibiting training-induced synergy carried mutations affecting *lpcA*, a gene in the lipopolysaccharide (LPS) core biosynthesis pathway. Among the five synergy-producing isolates, three harbored a large deletion encompassing *lpcA*, while the remaining two contained a nonsynonymous point mutation; no other genomic changes were consistently shared across these strains (Fig. 2a). Importantly, one synergy-producing isolate carried only an *lpcA* mutation and no other identifiable genomic changes, suggesting that *lpcA* disruption is responsible for the antibiotic sensitivity phenotype. For an additional line of evidence, we sought to directly test whether *lpcA* loss of function was sufficient to produce the observed increase in antibiotic sensitivity.

We measured the response of an *lpcA* knockout strain (ΔlpcA) from the Keio collection versus that of its isogenic K-12 parent to varying concentrations of erythromycin and rifampicin^35^. The ΔlpcA strain exhibited a consistent growth deficit compared to the WT strain, as indicated by lower optical density values recorded after 24 h of growth, which was consistent with Borin et al.’s previous findings of a fitness disadvantage borne by the strains with the *lpcA* mutations^18^ (Mann–Whitney *U* test: *U* = 8.000, *p* < 0.001) (Fig. 2b, c). This observation, although unrelated to antibiotic vulnerability, is in alignment with our overall hypothesis that trained resistance mutations can interfere with core cellular processes. Importantly, *lpcA* disruption is also associated with increased sensitivity to the antibiotic, where, across both erythromycin and rifampicin plots, we observe an earlier drop in optical density, or a lower concentration of antibiotic required to achieve 50% growth suppression (Fig. 2b, c; erythromycin: *U* = 1.500, *p* = 0.008; rifampicin: *U* = 0.000, *p* = 0.003). Because *lpcA* disruption was a striking feature shared across all isolates that exhibited training-induced synergy, and because its loss independently increased erythromycin and rifampicin sensitivity, these results indicate that training-induced synergy can arise through mutations affecting LPS core biosynthesis.

### Predicting two additional antibiotics capable of training-induced synergy

The observed correspondence between mutations associated with resistance to trained phages and the particular antibiotics that exhibited increased efficacy is consistent with known constraints on Gram-negative outer-membrane permeability. Erythromycin and rifampicin are large, hydrophobic antibiotics whose activity is strongly limited by lipopolysaccharide (LPS) integrity; resistance to these drugs often arises through exclusion rather than target modification^36^. Disruption of LPS core biosynthesis is therefore expected to disproportionately increase susceptibility to such antibiotics, whereas smaller or more polar antibiotics that enter cells through porins or active transport pathways should be less affected. This framework explains why training-induced collateral sensitivity emerged for a specific subset of antibiotics rather than producing broad-spectrum vulnerability.

To directly test this prediction, we repeated the IC50 assay using rifapentine and novobiocin, two antibiotics that are relatively large and hydrophobic. Rifapentine belongs to the same class as rifampicin, the rifamycins, which act by blocking bacterial transcription through the inhibition of DNA-dependent RNA polymerase^37^. Rifapentine and rifampicin are thus closely related in structure, with a few differences in certain functional groups. Novobiocin, on the other hand, belongs to the aminocoumarin class, unrelated to the rifamycins, exhibits a distinct structure, and functions by inhibiting DNA gyrase through a distinct mode of action^38^.

**Fig. 3:**
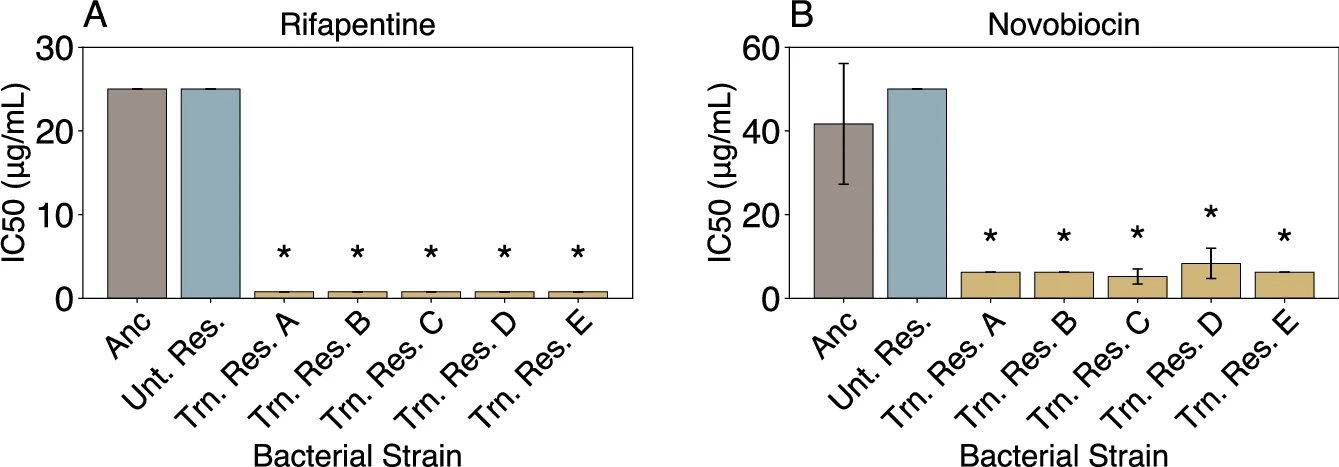
Predicted training-induced antibiotic synergies. IC50 values for rifapentine (**A**) and novobiocin (**B**) were measured for the λ-sensitive ancestral *E. coli* strain, a strain resistant to untrained phage λ, and multiple strains resistant to trained phage λ. In both panels, the ancestral and untrained-phage-resistant strains exhibited similarly high antibiotic resistance, whereas all trained-phage-resistant isolates showed significantly reduced IC50 values. Bars represent mean IC50 values, and error bars indicate standard deviations (*n* = 3 independent assays per strain). Asterisks denote statistically significant differences relative to the ancestral strain (rifapentine: Tukey’s post hoc test, *p* < 0.001; novobiocin: Tukey’s post hoc test, *p* < 0.001).

As expected, the ancestral strain and bacteria that evolved resistance to untrained phage λ exhibited comparably high resistance to rifapentine. In contrast, all isolates resistant to trained λ displayed significantly increased rifapentine sensitivity, with statistically indistinguishable IC50 values across isolates (Fig. 3a; one-way ANOVA: *p* < 0.001, no within-group variance observed; Tukey’s test). As for novobiocin, the same pattern was observed, where isolates resistant to the trained phage populations showed markedly lower IC50 values than the ancestral strain or the one that possessed resistance to the untrained phage population (Fig. 3b; one-way ANOVA: *p* < 0.001; Tukey’s test). Together, these results demonstrate that mechanistic insight into resistance pathways can be used to potentially predict novel phage-antibiotic synergies, highlighting a rational route for enhancing the therapeutic potential of phage training and a method for increasing chances of uncovering beneficial phage–bacterial pairs for evolutionary steering.

### Training in erythromycin alters phage suppressiveness

We next asked whether conducting coevolutionary phage training in the presence of erythromycin influences the suppressive ability of the resulting phages. The observed synergies suggested that antibiotics may impose fitness costs on bacterial mutations, thereby biasing which resistance strategies emerge, which counter-resistance mechanisms phages evolve in response, and how these interactions unfold over time. By constraining some evolutionary routes while favoring others, antibiotics could redirect coevolutionary trajectories toward distinct phenotypic endpoints. If so, training phages in the presence of antibiotics may yield phages better adapted to clinical environments, where bacteria experience ongoing pharmacological pressure.

To test this, we conducted a 21-day coevolutionary training experiment comparing phages evolved in the presence of erythromycin with phages evolved under standard training conditions without antibiotics. We focused on erythromycin rather than rifampicin because preliminary coevolutionary training trials with rifampicin produced inconsistent and non-reproducible outcomes. We also did not use rifapentine or Novobiocin because, at the time of this study, we had yet to test the effects of them. At the end of training, we isolated one phage from each of the six replicate populations from each treatment for downstream comparison. We then quantified the suppressive ability of each trained phage isolate using 7-day suppression assays, in which bacteria are challenged with a single phage isolate and population density is measured over time to assess how effectively bacterial growth is constrained. Suppression assays were performed against the ancestral *E. coli* REL606 strain in both the presence and absence of erythromycin.

**Fig. 4:**
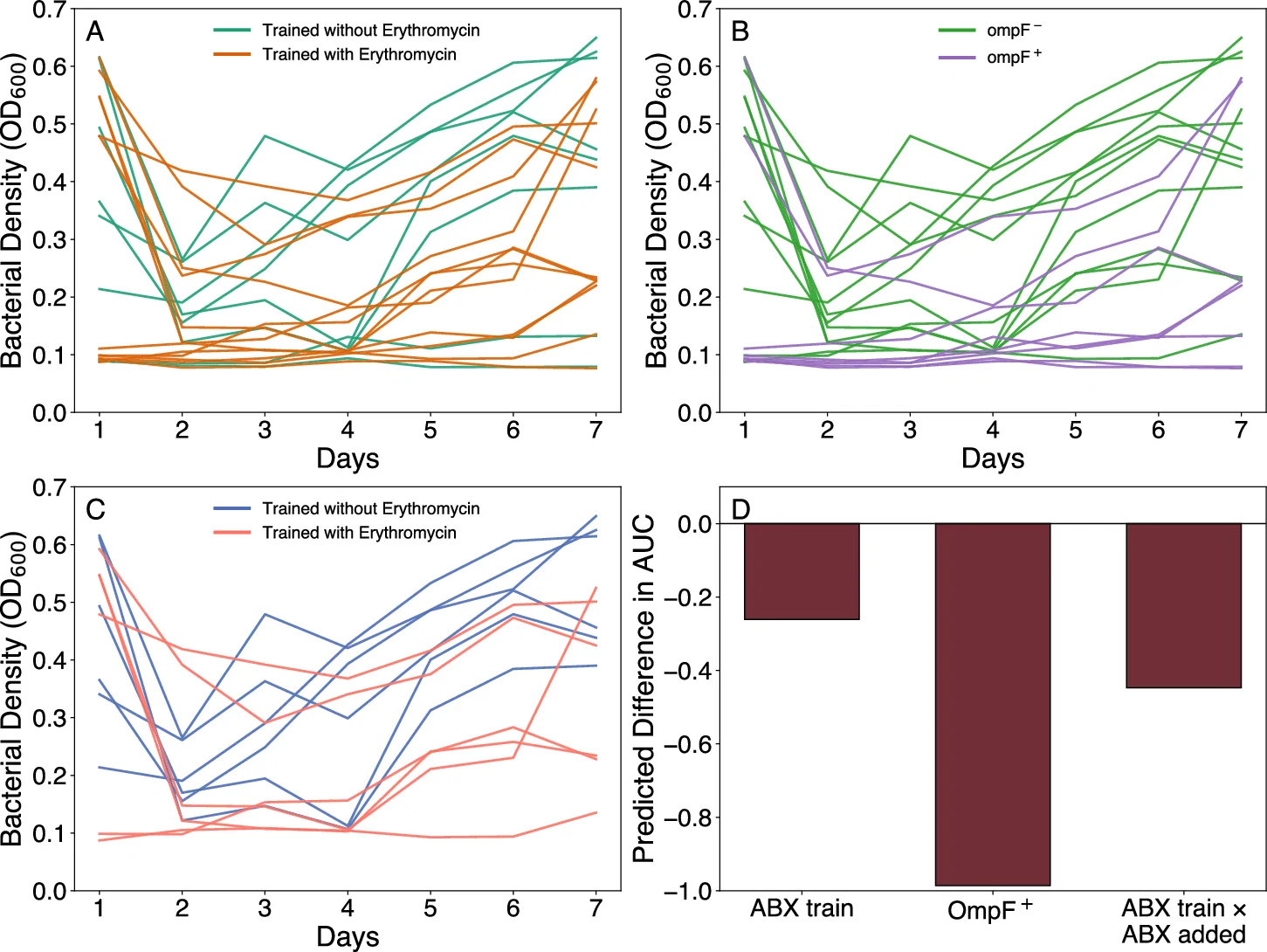
Bacterial suppression assays comparing phages evolved under different training conditions (*n* = 3 biological replicates per treatment). For panels **A**–**C**, bacterial density was measured over time, with lower densities indicating greater suppression. **A** Time-course suppression assay comparing phages trained in the presence of erythromycin with phages trained without antibiotics. **B** Suppression assays comparing phages that evolved the ability to use OmpF with phages that did not. **C** Suppression assays restricted to LamB-reliant phages only, comparing erythromycin-trained and no-antibiotic-trained phages. **D** Residuals from the area-under-the-curve (AUC) analysis corresponding to panels (**A**–**C**). AUC values summarize bacterial density over the full time course, with lower AUC values indicating stronger suppression; residuals represent deviations from the fitted model used to compare treatments.

On average, phages trained in erythromycin exhibited greater overall suppression of bacterial growth than phages from no-antibiotic training (*p* = 0.044; Supplementary Table 1). However, this increased suppressiveness was not further enhanced when assays were conducted in the presence of antibiotics, indicating that the effect was general rather than antibiotic specific as our hypothesis predicted (Fig. 4, Supplementary Fig. 1; Supplementary Table 1). This pattern led us to ask whether a correlated trait, rather than antibiotic-specific adaptation per se, underlies the increased suppression observed among phages trained with antibiotics.

Previous work has shown that some phages evolve during training to infect via two receptors—LamB (native) and OmpF (novel)—and that these dual-receptor (OmpF⁺) phages exhibit superior suppressive ability relative to LamB-restricted phages^18^. Consistent with this, erythromycin training produced OmpF⁺ phages more frequently than standard training (three independent populations versus one). To test whether this difference in OmpF⁺ frequency accounts for the apparent treatment effect, we fit a multiple linear model using R that included antibiotic training (with or without antibiotics), assay environment (with or without antibiotics), their interaction, and phage OmpF-use status as predictors of suppression (AUC). In this model, only OmpF-use significantly explained variation in AUC, indicating that enhanced suppression is driven by receptor usage rather than antibiotic-specific training effects (Fig. 4b–d; Supplementary Table 2).

**Fig. 5:**
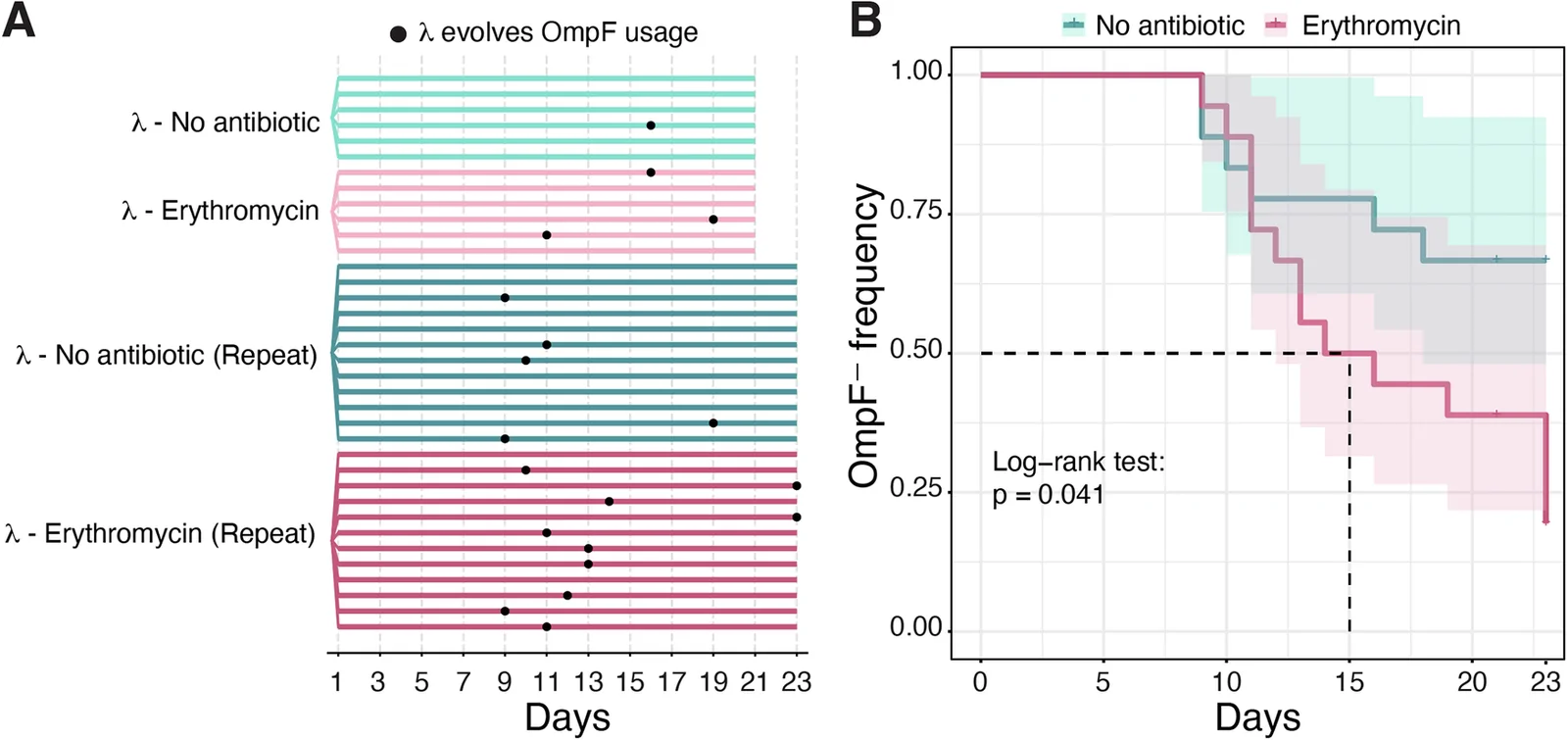
OmpF+ evolutionary dynamics. **A** Evolution of OmpF usage during 21-day coevolutionary training experiment (*n* = 6) and extended 23-day repeat (*n* = 12). Each line represents a separate evolutionary trial. **B** Kaplan–Meier-style analysis showing a statistically significant decline in OmpF^−^ phage frequency in the erythromycin-trained group relative to the no-antibiotic group. Shaded regions represent 95% confidence intervals.

Next, we asked whether the higher frequency of OmpF⁺ phage populations observed during erythromycin training reflects a causal effect of antibiotics on phage evolution. Specifically, could the presence of antibiotics alter the coevolutionary dynamics in a way that favors the evolution of OmpF usage? Since the initial observation—1 of 6 OmpF⁺ populations in the no-antibiotic treatment versus 3 of 6 in the erythromycin treatment—was not statistically significant, we repeated coevolutionary training in the presence and absence of erythromycin with a larger cohort (12 replicates) and tracked the timing of OmpF acquisition over 23 days, allowing us to test whether OmpF usage evolves more frequently and/or more rapidly under antibiotic exposure. In this expanded dataset, 5 of 12 phage populations evolved OmpF usage under no-antibiotic training, compared with 10 of 12 trained with erythromycin (Fig. 5a).

To statistically evaluate differences in the rate of OmpF acquisition, we adapted a Kaplan-Meier survival analysis, commonly used to model time-to-event data in clinical studies. Here, the “event” corresponds to the gain of OmpF usage rather than mortality. This analysis revealed a significantly faster decline in the fraction of OmpF^−^ phages in the erythromycin-trained populations relative to the no-antibiotic controls (*p* = 0.041), indicating that phages trained in the presence of erythromycin evolved OmpF usage both more frequently and more rapidly (Fig. 5b).

Together, these findings suggest that erythromycin training does not produce phage strains that are better adapted to suppress bacteria in the presence of antibiotics, but instead increases suppressiveness specifically by supporting the emergence of OmpF gain of function. Thus, the antibiotic environment modifies coevolutionary trajectories in a manner that promotes this beneficial receptor innovation.

### Training in erythromycin is associated with *manXYZ* mutation suppression in *E. coli*, encouraging OmpF innovation

How might erythromycin alter coevolutionary dynamics to favor the evolution of OmpF usage? One possibility is suggested by prior work characterizing coevolution between *E. coli* REL606 and phage λ^39^. In that system, bacterial resistance commonly arises through *malT* mutations, which reduce LamB expression. By making the native receptor rare on the cell surface, these mutations favor selection on λ’s host-recognition protein (J) to increase adsorption efficiency^40^. Under this regime, a rare but fortuitous combination of multiple *J* mutations can allow phage λ to exploit a novel receptor, OmpF. However, if bacteria subsequently acquire resistance through mutations in *manXYZ*, which encodes the inner-membrane transporter required for phage DNA entry, selection shifts away from *J* and toward other phage components, such as the tail protein H, that counter this second line of defense^41,42^. Once this shift occurs, the phage no longer accumulates the necessary constellation of *J* mutations, and the OmpF-use innovation fails to arise^39^. This framework led us to hypothesize that erythromycin exposure may reduce the rate at which *manXYZ* resistance evolves, thereby prolonging the period during which selection acts on *J* and increasing the probability of OmpF-use acquisition.

**Fig. 6:**
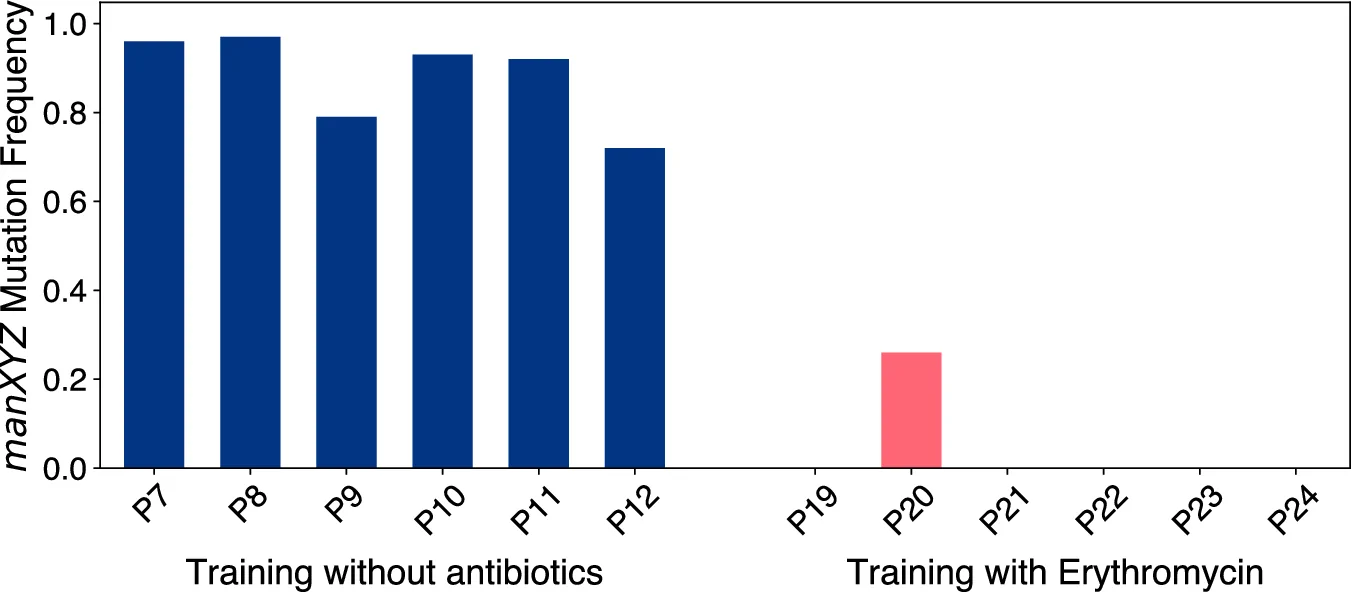
*manXYZ* resistance mutations rise differs by treatment. Frequency of *manXYZ* mutants across replicate bacterial populations co-occurring with phage on the 21st day of training with and without erythromycin (*n* = 6 distinct replicates per treatment). Missing values indicate no *manXYZ* mutants were observed.

To test this hypothesis, we isolated bacterial populations from day 21 of the initial training experiment and screened for *manXYZ* mutations using tetrazolium–mannose indicator plates, which distinguish *manXYZ* mutants based on colony color^41^. We then quantified mutation frequencies from colony counts. Host populations evolved in the presence of erythromycin exhibited markedly lower frequencies of *manXYZ* mutations than those evolved without antibiotics (Mann–Whitney *U* test: *U* = 36.0, *p* = 0.004; Fig. 6), consistent with erythromycin constraining this resistance pathway.

This reduction in *manXYZ* mutation frequency in the erythromycin treatment suggests that, for reasons not yet clear, antibiotics appear to suppress bacterial host escape routes that would otherwise block OmpF-use evolution, extending the window to evolve the ability to use this receptor. To explore whether erythromycin imposes subtle fitness costs that might disfavor *manXYZ* mutants, we compared the fitness of host populations in the presence and absence of erythromycin. However, these preliminary analyses of growth costs in the presence of erythromycin revealed substantial variability and no statistically significant differences between the erythromycin and no-antibiotic groups (Supplementary Fig. 2). We also directly measured erythromycin sensitivity in *manY* and *manZ* knockout strains, and this assay likewise showed no increased susceptibility that could account for the reduced frequency of *manXYZ* mutations (Supplementary Fig. 3). Therefore, although the decrease in *manXYZ* mutations offers insight into the mechanistic basis of how erythromycin encourages OmpF innovation and enhances suppression for trained phages, the underlying reasons behind this phenomenon are unknown and are suspected to be driven by more complex eco-evolutionary interactions within the phage-bacterium ecological community and arms race.

## Discussion

A central finding of this study is that resistance to a trained, but not an untrained, phage λ generates increased sensitivity to erythromycin, rifampicin, novobiocin, and rifapentine, providing direct evidence that coevolutionary phage training itself can produce phage–antibiotic synergy. Classical phage-antibiotic synergy arises when resistance to a phage incidentally compromises bacterial physiology, increasing susceptibility to antibiotics. Consistent with this framework, we observed such “typical” synergy with streptomycin regardless of phage training history. In contrast, synergy with erythromycin, rifampicin, novobiocin, and rifapentine emerged only following resistance to trained phage λ, indicating that this outcome depends on how resistance evolves, rather than on phage resistance per se.

We propose that training-induced phage-antibiotic synergy arises because coevolutionary training biases bacterial evolution toward resistance mutations that are effective against phage but carry substantial pleiotropic costs. Consistent with this, resistance to trained phage λ frequently involved mutations disrupting *lpcA*, a gene implicated in LPS core biosynthesis. Disruption of *lpcA* produces LPS phenotypes that compromise outer-membrane integrity and fundamentally alter permeability^36,43^. Independent isolates resistant to trained phage λ and exhibiting increased sensitivity to erythromycin and rifampicin all carried *lpcA* disruptions, and loss of *lpcA* alone was sufficient to recapitulate this phenotype in an isogenic background. Together, these results identify LPS core disruption as a key functional link between resistance to trained phage λ and increased antibiotic susceptibility.

Importantly, this effect was not universal across antibiotics: only two of the seven original drugs screened exhibited training-induced synergy. This drug specificity suggests that training-induced sensitivity reflects targeted alterations to the bacterial envelope rather than generalized physiological decline. In particular, the observed pattern aligns with known constraints on Gram-negative outer-membrane permeability. Erythromycin and rifampicin are large, hydrophobic antibiotics whose activity is strongly limited by LPS integrity; resistance often arises through exclusion rather than target modification^36^. Disruption of LPS core biosynthesis is therefore expected to disproportionately increase susceptibility to these drugs, whereas smaller or more polar antibiotics that enter through porins or active transport pathways would be less affected. To test this, we identified two other drugs, novobiocin and rifapentine, with these qualities and found that they too showed the pattern of training-induced collateral-sensitivity, providing additional evidence that the LPS mutations mediated the antibiotic-trained phage resistance interaction.

Notably, LPS disruption seems to be a repeated outcome of selection with trained λ. Previous work on trained λ identified additional resistant mutants lacking *lpcA* mutations, but they carried other alterations predicted to affect LPS structure or biosynthesis, suggesting convergence at the level of cellular function despite genetic heterogeneity^18^. Additionally, previous studies have shown collateral sensitivity between phages and antibiotics mediated by LPS mutations^25^. Together, these results indicate that coevolutionary phage training can reliably bias resistance evolution toward LPS modification, thereby creating predictable, drug-specific antibiotic vulnerabilities.

Another important point of discussion arising from our observations was that introducing erythromycin into the phage-training environment revealed that antibiotics can act as powerful modifiers of phage-host coevolution, in ways that are not readily predicted from their direct antimicrobial effects. Rather than producing phages that were specifically optimized for antibiotic-containing environments, erythromycin altered the structure of the evolutionary landscape itself, biasing which bacterial escape routes were accessible and, in turn, which phage adaptations could emerge. The net result was the evolution of phages with enhanced suppressive ability across environments, an outcome that was both indirect and unexpected.

More broadly, these findings highlight that antibiotics can influence phage evolution not only by imposing additional stress on bacteria but by reshaping the coevolutionary pathways available during training. By constraining certain resistance strategies, antibiotics can prolong or redirect selective pressures on phages, increasing the likelihood of otherwise rare innovations with large phenotypic consequences. At the same time, the direction of this effect is not guaranteed: under different ecological, genetic, or pharmacological conditions, antibiotics could instead restrict adaptive routes available to phages, slow evolutionary responses, or favor bacterial resistance pathways that limit phage innovation. Which outcome prevails will depend on the specific interactions among antibiotics, bacterial resistance mechanisms, and phage counteradaptations, emphasizing the inherently path-dependent nature of phage-bacteria-antibiotic evolution.

From a therapeutic perspective, these results suggest that phage training in the presence of antibiotics may sometimes be leveraged to bias phage evolution toward more broadly suppressive or robust phenotypes, even when no direct phage-antibiotic synergy is observed during treatment. However, we must caution that we do not yet know how universal this effect is and that poorly chosen conditions could have the opposite effect, reducing phage evolvability or therapeutic potential. Understanding when antibiotics accelerate, redirect, or suppress phage evolution will therefore require systematic exploration across drugs, hosts, and phage systems. Viewed through this lens, our results underscore the importance of higher-order evolutionary interactions and historical contingency in shaping outcomes in phage-bacteria-antibiotic systems.

Overall, our results suggest that phage therapy design may benefit from explicitly accounting for evolutionary dynamics that unfold during phage preparation, not just during clinical deployment. Coevolutionary training can bias bacterial resistance toward costly pathways that increase antibiotic sensitivity, while antibiotics present during training can reshape coevolutionary trajectories in ways that promote phage innovations associated with enhanced suppressiveness. Importantly, these effects arise through indirect, higher-order interactions rather than simple additive antimicrobial activity.

By showing that coevolutionary training can channel bacterial resistance toward predictable molecular vulnerabilities—and that antibiotics can be selected to exploit those vulnerabilities—we provide a strategy for engineering synergy from both sides of the phage–antibiotic interaction. This work moves phage–antibiotic therapy beyond opportunistic pairing toward a principled, evolution-informed design paradigm.

## Methods

### Media

All bacterial and phage experiments were conducted using the following media formulations. LB Lennox medium consisted of 20 g/L Lennox powder (premixed tryptone, yeast extract, and NaCl) dissolved in water. Soft agar overlays were prepared using 10 g tryptone, 5 g yeast extract, 5 g NaCl, and 7 g agar per liter of water. Tris-DM glucose medium was prepared by combining 50 mL of 1 M Tris-HCl (pH 7.4), 0.28 g K₂HPO₄, 0.08 g KH₂PO₄, and 1 g ammonium sulfate in 1 L of water; after sterilization, 5 mL of 20% glucose, 1 mL of 20% thiamine, 200 µL of 1 M CaCl₂, 200 µL LB Lennox, and 10 µL of 1 M MgSO₄ were added. M9-glucose medium consisted of 200 mL M9 medium, 10 mL of 20% glucose, 1 mL of 0.2% thiamine, 200 µL of 1 M CaCl₂, and 200 µL LB diluted to 1 L with water. Tetrazolium mannose plates for detecting *manXYZ* mutants were prepared by dissolving 10 g tryptone, 1 g yeast extract, 5 g NaCl, and 16 g agar in 800 mL of water and combining this with a separate solution of 10 g mannose in 200 mL water; after sterilization and cooling, 1 mL of 5% TTC (2,3,5-triphenyltetrazolium chloride) was added before pouring. Plates were left covered at room temperature for 3-5 days prior to use. 0.2 mg/mL (200 μg/mL) stocks of the following antibiotics were made and stored in a −20 °C freezer: kanamycin, gentamicin, carbenicillin, and streptomycin were dissolved in molecular grade water, erythromycin and chloramphenicol were dissolved in 95% ethanol, and rifampicin and ciprofloxacin were dissolved in DMSO (dimethyl sulfoxide).

### IC50 antibiotic susceptibility assays

The strains used for this experiment were obtained from Borin et al.’s work in 2021, but were renamed to be more descriptive and better fit the focus of this study^18^. Our Resistant to Untrained λ strain (Untr. Res.) was renamed from λunt P1-T3-2R, containing one mutation, and was obtained from their coevolutionary training experiment. Our Resistant to Trained λ strains (Trn. Res.) A and B correspond to λtrn P2-T25-9-R and P2-T25-10-P, which carry 24 and 5 mutations, respectively, with full and partial resistance to λtrn. Given the large number of mutations and the nature of the experiment, not all mutations necessarily evolved for resistance. Trn. Res. C corresponds to JB42-P, harboring a single mutation conferring partial resistance to the trained phage. Trn. Res. D refers to JB43-P and contains three mutations, also resulting in partial resistance. Trn. Res. E corresponds to JB47-R with two mutations that result in complete resistance to the trained phage. Trn. Res. C-E were obtained from their Luria-Delbrück experiments, and most, if not all, mutations must contribute to resistance. They all exhibit at least one mutation in the *lpcA* gene, with Trn. Res. C not bearing any additional alterations (see Fig. 2a; Supplementary information^18^).

Antibiotic susceptibility was quantified by measuring the half-maximal inhibitory concentration (IC50) for each strain across seven antibiotics, in addition to novobiocin and rifapentine. For each antibiotic, three independent 96-well plates were prepared, each containing 1 trial for each strain. Strains were revived from frozen stocks and grown overnight in LB Lennox at 37 °C with shaking at 220 rpm. Overnight cultures were diluted to approximately 10⁴ CFU/mL in fresh LB Lennox and used immediately for inoculation.

Antibiotic gradients were generated in columns 2–10 of each plate. Column 10 contained 200 μg/mL of the respective antibiotic, and twofold serial dilutions were performed through column 2, reaching a final concentration of 0.78 μg/mL. Column 1 contained no antibiotic (growth control), column 11 contained antibiotic only (no-cell control), and column 12 contained LB Lennox only (media blank). In all wells, 100 µL of antibiotic solution (or LB Lennox for controls) was combined with 100 µL of diluted culture, yielding a final volume of 200 µL per well and halving the previously obtained antibiotic concentrations.

Plates were incubated statically at 37 °C for 24 h. Optical density at 600 nm (OD₆₀₀) was measured at the 24-h endpoint using a Tecan Sunrise plate reader. For each strain-antibiotic combination, IC50 values were calculated from OD₆₀₀ measurements by first defining the maximal and minimal growth observed for that strain. The half-maximal OD₆₀₀ value was calculated as the midpoint between these maxima and minima, and the corresponding antibiotic concentration was taken as the IC50. When the half-maximal value fell between two tested concentrations, the higher concentration was assigned to provide a conservative estimate.

### Assessing Sensitivity of the *lpcA* Knockout to Erythromycin and Rifampicin

To determine whether loss of *lpcA* function is sufficient to increase sensitivity to erythromycin and rifampicin, we compared the antibiotic responses of an *lpcA* knockout strain (ΔlpcA) from the Keio collection with its isogenic wild-type parent *E. coli* K-12 BW25113^35^. Both strains were revived from frozen stocks and grown overnight in LB Lennox at 37 °C with shaking at 220 rpm. Cultures were then diluted to approximately 10⁴ CFU/mL in fresh LB Lennox.

Antibiotic assays were then performed in 96-well plates identically as the aforementioned IC50 assays, but with antibiotic concentrations ranging from 0.1 to 100 μg/mL, including a column with 0 μg/mL. In total, six replicates were obtained.

### Coevolutionary phage training in the presence and absence of erythromycin

Coevolutionary training experiments were conducted using *E. coli* B REL606 as the bacterial host, bacteriophage λ cI26 as the ancestral phage, and 1.56 µg/mL erythromycin as the antibiotic treatment. Four experimental conditions were established: (i) bacteria only, (ii) bacteria + phage (typical training), (iii) bacteria + erythromycin, and (iv) bacteria + phage + erythromycin (antibiotic training). Each condition was run in 50 mL Erlenmeyer flasks containing 10 mL M9 glucose minimal medium supplemented with 100 µL of 1 M MgSO₄.

For treatments including bacteria, 100 µL of REL606 diluted to ~10⁴ CFU/mL in LB Lennox was added to each flask. For treatments including phage, 100 µL of λ cI26 diluted to ~10³ PFU/mL in Tris–LB was added. All flasks were incubated at 37 °C with shaking at 120 rpm for 24 h. Each subsequent day, 100 µL of the prior day’s culture was transferred into a fresh flask containing 10 mL M9 glucose, 100 µL MgSO₄, and erythromycin where applicable. Cultures were again incubated under identical conditions for an additional 24 h. This serial-passage procedure was repeated. Initially, the experiment was performed with 6 biological replicates for 21 days; however, it was repeated with 12 replicates for 23 days to better study the emergence of OmpF usage.

Daily phage isolates were collected by removing 1 mL of culture, centrifuging at 15,000 rpm for 1 min, and filtering the supernatant through 0.22-µm Millex-GP filters. Filtrates were serially diluted 10-fold in M9 glucose (to 10^−^⁷), and 2-µL spots of each dilution were plated onto bacterial lawns to quantify titers and assess receptor usage. To archive daily phage populations, 170 µL of lysate was mixed with 30 µL 80% v/v glycerol in 96-well plates and stored at −80 °C.

Bacterial lawns were prepared by mixing soft agar with 250 µL of bacteria and pouring onto 150 mm × 15 mm plates. Two host genotypes were used: REL606 (expressing LamB and OmpF) to detect general infectivity, and strain 19a, a LamB-knockout REL606 derivative, to detect phages capable of infecting via a secondary receptor^44^. Plates were incubated overnight at 37 °C to track plaquing patterns and determine the daily presence or absence of phages infecting hosts lacking LamB.

### Suppression assays

To evaluate the suppressive ability of trained phages, we performed serial-passage suppression assays using *E. coli* B REL606 as the host. Phages were first amplified overnight on REL606 and isolated by centrifugation at 3900 rpm for 10 min, followed by filtration through 0.22-µm filters. Phage lysates were diluted to approximately 10⁴–10⁵ PFU/mL in Tris–LB. REL606 was grown overnight and diluted to ~10³ CFU/mL in LB Lennox.

Suppression assays were initiated in 20 × 150 mm glass culture tubes with three biological replicates per treatment (with and without erythromycin). Each tube contained 4 mL M9 glucose medium, 40 µL of 1 M MgSO₄, 100 µL of diluted REL606 (~10³ CFU/mL), and 10⁴–10⁵ phage particles. For the erythromycin treatment, tubes were supplemented with erythromycin to a final concentration of 1.56 µg/mL, identical to the concentration used during coevolutionary training. Tubes were incubated at 37 °C with shaking at 220 rpm for 24 h.

Each day, 40 µL of the previous day’s culture was transferred into fresh M9 glucose medium with MgSO₄, with erythromycin added where appropriate, and cultures were incubated under the same conditions. To quantify bacterial density over time, 200 µL from each replicate was transferred daily into two separate 96-well plates, and optical density was measured using a Tecan Sunrise plate reader. After OD measurement, plates were sealed with sterile adhesive foil and centrifuged at 3900 rpm for 3 min.

To monitor phage infectivity, 2-µL aliquots from each lysate were spotted onto REL606 lawns prepared on LB agar plates. Lysates were also archived daily by mixing 170 µL of culture with 30 µL of 80% (v/v) glycerol in 96-well plates and storing the plates at −80 °C.

### *ManXYZ* mutation detection

To determine the frequency of *manXYZ* mutations in host populations from the coevolutionary training experiment, we isolated bacteria from the frozen Day 21 endpoint samples. For each replicate population, a small amount of frozen culture was obtained by freezer scraping and resuspended in Tris–DM glucose, the same medium used during coevolution. Samples were serially diluted to approximately 10⁶ CFU/mL, and 100 µL of the final dilution was spread onto tetrazolium mannose indicator plates, which enable phenotypic detection of *manXYZ* mutants^41^.

Plates were incubated overnight at 37 °C. Colonies exhibiting a red phenotype were scored as *manXYZ* mutants, whereas white or pale-pink colonies were scored as wild-type for the *manXYZ* locus^41^. Mutation frequency was calculated as the proportion of red colonies relative to the total colony count for each replicate population.

### Statistical analysis

All statistical analyses were two-tailed with *α* = 0.05 when unspecified. Data are reported with *n*, a measure of central tendency, and variability as specified in the figure legends; error bars denote standard deviation unless otherwise indicated. IC50 values were compared within each antibiotic using one-way ANOVA followed by Tukey’s HSD for multiple comparisons. Nonparametric Mann–Whitney *U* tests were used for pairwise comparisons involving small sample sizes or non-normal data distributions. Bacterial suppression was summarized as the area under the curve (AUC) and analyzed using multiple linear models, including training condition, assay environment, their interaction, and phage receptor usage. The standard R package was used for the analyses (R 4.5.2). Model assumptions were verified by inspection of residuals. Statistics were run using Python^45^. Time to acquisition of OmpF usage was analyzed using Kaplan-Meier survival analysis with log-rank tests. R package “survival” (https://cran.r-project.org/web/packages/survival/index.html) was used to perform the survival analysis, and the package “ggplot2” to plot survival curves (https://ggplot2.tidyverse.org/)^46^.

## Supplementary information

Supplementary Information

## Data Availability

Experimental data are archived and freely available at Dryad; https://doi.org/10.5061/dryad.7m0cfxqbt.
