# Supplementary material for: Combining Evolutionary Steering and Coevolutionary Phage Training to Generate Predictable Phage-Antibiotic Synergy
Source: NPJ Antimicrob Resist. 2026 Jul 14;4:78. doi: 10.1038/s44259-026-00249-w (PMC13745444; doi:10.1038/s44259-026-00249-w)
Supplement: Supplementary file 1 [file 44259_2026_249_MOESM1_ESM.docx]

## **Supplementary Information for**

**Combining Evolutionary Steering and Coevolutionary Phage Training to Generate Phage-Antibiotic Synergy**

Nehme Lahoud^1^, Sweetzel D. Labador^1^, Mrudula Sane^1^, Divya Ram^1^, Gabriel E. Diaz^1^, Justin R. Meyer^1*^

1: Department Ecology, Behavior and Evolution, University of California San Diego, San Diego, CA 92093

*: jrmeyer@ucsd.edu

**Suppression Experiment Populations**

**Supplementary Figure 1.** Individual populations during the suppression assays grouped by training method (erythromycin vs typical) and by OmpF usage status.

**AUC Statistical Analysis**

**Supplementary Table 1.** Comparison of statistical models predicting bacterial suppression that vary in complexity and explanatory structure. Models range from a simple comparison of suppression (area under the curve, AUC) between phages trained with antibiotics and those trained without antibiotics, to progressively more complex models that incorporate suppression measured in the presence of antibiotics and interaction terms between training condition and assay environment. Interaction terms test whether antibiotic training confers suppression that is specific to antibiotic-containing conditions. The final model includes phage OmpF usage as an additional predictor and provides the best overall fit, indicating that receptor usage explains more variation in suppression than antibiotic-specific training effects.

**Supplementary Table 2.**
Summary statistics for the best-performing model predicting bacterial suppression. Phage OmpF usage is the strongest predictor of suppression. Although antibiotic training is significant in simpler models, this effect is explained by the higher frequency of OmpF-using phages in the antibiotic-trained treatment, rather than by antibiotic-specific adaptations that directly enhance suppression.

***ManXYZ* Competition Experiment**

We directly tested the hypothesis that *manXYZ* mutations incur an additional fitness cost in the presence of erythromycin. To do so, we isolated *manXYZ*-positive and *manXYZ*-negative clones from each population evolved without antibiotics (because *manXYZ⁻* genotypes did not evolve consistently under antibiotic treatment). We then competed matched pairs of these clones under the same experimental conditions used during the coevolutionary training assays, with the sole modification that one set of competitions included erythromycin.

We quantified changes in relative clone frequencies over a 24-hour competition and computed relative fitness as the ratio of exponential growth rate of *manXYZ⁻* divided by the exponential growth rates*manXYZ⁺*. Six independent *manXYZ⁻*/ *manXYZ⁺* pairs were tested, with one competition per pair per condition.

Contrary to our prediction, we observed a slight, non-significant fitness advantage of *manXYZ* mutants in the presence of erythromycin. Given the lack of support, we did not pursue this hypothesis beyond this pilot experiment.

**Supplementary Figure 2.** Relative fitness of *manXYZ* minus isolates compared to coexisting *manXYZ* positive isolates in two environments, with and without Erythromycin. Error bars represent 95% confidence intervals.

**Sensitivity of ManY and ManZ Knockouts to Erythromycin**

We performed an erythromycin IC50 analysis with Δ*manY*, Δ*manZ*, and WT strain and found no significant differences in sensitivity to erythromycin between the knockouts and WT. Both knockouts were obtained from the Keio collection and the wild-type strain used was *E. coli* K-12 BW25113^1^.

**Supplementary Figure 3.** Sensitivity of *manY* and *manZ* knockouts, as well as WT isolates to erythromycin.

**SI References:**

1.	Baba, T. *et al.* Construction of Escherichia coli K-12 in-frame, single-gene knockout mutants: the Keio collection. *Mol. Syst. Biol.* **2**, 2006.0008 (2006).
